# Implantation of Impella CP left ventricular assist device under the guidance of three-dimensional intracardiac echocardiography

**DOI:** 10.1038/s41598-020-74220-8

**Published:** 2020-10-15

**Authors:** Konstantin Yastrebov, Laurencie Brunel, Hugh S. Paterson, Zoe A. Williams, Innes K. Wise, Christopher S. Burrows, Paul G. Bannon

**Affiliations:** 1 Sydney Imaging, Core Research Facility, The University of Sydney, Sydney, Australia; 2 The Baird Institute of Applied Heart and Lung Surgical Research, Sydney, Australia; 3 Institute of Academic Surgery, Royal Prince Alfred Hospital and University of Sydney, Sydney, Australia; 4 Prince of Wales Hospital, Sydney, University of New South Wales, Barker St, Randwick, 2031 Australia

**Keywords:** Cardiac device therapy, Translational research

## Abstract

Impella CP is a percutaneously inserted left ventricular assist device indicated for temporary mechanical cardiac support during high risk percutaneous coronary interventions and for cardiogenic shock. The potential application of Impella has become particularly relevant during the current COVID-19 pandemic, for patients with acute severe heart failure complicating viral illness. Standard implantation of the Impella CP is performed under fluoroscopic guidance. Positioning of the Impella CP can be confirmed with transthoracic or transoesophageal echocardiography. We describe an alternative approach to guide intracardiac implantation of the Impella CP using two-dimensional and three-dimensional intracardiac echocardiography. This new technique can be useful in selected groups of patients when fluoroscopy, transthoracic and transoesophageal echocardiography is deemed inapplicable or limited for epidemiological or clinical reasons. Intracardiac three-dimensional echocardiography is a feasible alternative to the traditional techniques for implantation of an Impella CP device but careful consideration must be given to the potential limitations and complications of this technique.

## Introduction

Impella CP is a microaxial pump left ventricular assist device licensed for use for several hours during high risk percutaneous coronary interventions and up to four days in patients with cardiogenic shock. It has recently been given emergency approval by the United States Food and Drug Administration for use in COVID-19 patients with extracorporeal membrane oxygenation support. Successful clinical off-label use for several weeks has been previously reported. Other models of Impella (Impella 5.0 and 5.5) are licensed for left ventricular support up to 14 days. The purpose is to improve cardiac output sufficiently to prevent secondary organ failure and to unload left ventricle. Among other indications, Impella has been used in acute viral myocarditis^1,2^. The standard technique for the implantation of Impella CP involves insertion of the percutaneous femoral artery introducer for the guidewire to be advanced via the aorta and aortic valve into the left ventricle under fluoroscopy. The microaxial pump is then implanted using modified Seldinger technique. The blood inlet of the device is positioned within left ventricular cavity 3.5 cm below the aortic valve and an outlet together with the 14Fr pump motor—above the aortic valve. A 9Fr reinforced catheter traverses the aortic valve to provide continuous flow of up to 3.7 l/min of blood from the left ventricle into systemic circulation. Correct positioning of the Impella is paramount for optimization of flow and avoidance of complications. Of the particular importance is the position of the blood inlet which may inadvertently impinge on the anterior mitral leaflet, mitral subvalvular apparatus, left ventricular wall and left ventricular outflow tract walls, and at times migrate into aortic root. Transthoracic (TTE) and transesophageal (TEE) echocardiography is used to confirm position^3^. Adequate echocardiographic imaging with transthoracic echocardiography in intensive care patients is not always possible because of overdistended lungs by positive pressure ventilatory support, non-optimal patient’s positioning and postoperative and posttraumatic chest complications. Transesophageal echocardiography in hypoxemic patients requires endotracheal intubation. Several co-existing pathologies can constitute contraindication for TEE.

The initial data from COVID-19 infected patients suggests that up to 23% of patients develop a degree of myocardial injury, while up to 40% of deaths have been attributed to cardiac failure, most likely a result of viral myocarditis^4^. Impella left ventricular support devices have been successfully used in the temporary mechanical circulatory support of patients with myocarditis, making it a potential option for SARS CoV2 patients with severely deteriorating cardiac failure. Recent recommendations from the Cardiac Society of Australia and New Zealand (CSANZ)^5^ and the American Society for Artificial Internal Organs (ASAIO)^6^ suggested that Impella and veno-arterial extracorporeal oxygenation (VA ECMO) should be considered alone or in combination (ECPELLA) for COVID-19 patients suffering acute cardiac failure. Further, clinical reports are now emerging from the American College of Cardiology^7^ Massachusetts General Hospital^8^ and Northwestern Medicine^9^ regarding the use of Impella in patients suffering from cardiogenic complications of COVID-19.

The use of fluoroscopy in this group of patients requires transfer to the catheterization laboratory or radiology department and represents a significant risk for unstable and highly infectious patients, staff and other patients within the hospital. The use of TTE and TEE in this subgroup of patients will be difficult due to the routine use of high positive end expiratory pressure and the need for intubation in patients who are non-invasively ventilated via a face mask or a helmet.

Two-dimensional and three-dimensional intracardiac echocardiography (3D ICE) is an invasive procedure where a catheter is introduced via a femoral or jugular vein into the right heart chambers. The tip of the catheter is equipped with ultrasound emitting and receiving elements able to provide two and three-dimensional grey-scale imaging, two and three-dimensional Colour Doppler and spectral Doppler interrogation. A range of frequencies up to 8 MHz in fundamental and harmonic modes offers good axial resolution due to the intimate proximity to the cardiac structures. Recent technological advances allow instantaneous three-dimensional imaging with up to 20 volumes per second with a wide imaging sector of 90° azimuthal and elevation of up to 50°. A previously available narrow-angle (up to 24° elevation) 3D ICE suffered from an inability to capture sufficient volumes to be useful in assessing whole valves or spatial relationships between cardiac structures and intracardiac devices. Wide-angle 3D imaging largely rectified this limitation, offering a potential alternative approach for Impella implantation when standard guidance techniques are not clinically applicable, including for selected subgroups of COVID-19 patients.

We hypothesised that it is feasible to conduct implantation of the Impella CP using intracardiac two-dimensional and three-dimensional echocardiographic guidance without the need for fluoroscopy of other echocardiographic imaging.

## Results

Eight adult first cross merino ewes were used for 25 experimental implantations of the Impella CP. Sheep characteristics are presented in Table E1. Haemodynamic variables prior to the implantation of the Impella CP are presented in Table E2.

### Initial position guidance

Quality of 3D ICE imaging of the relevant anatomical structures for each sheep is presented in Table 1. Quality of 3D ICE imaging of the procedural components for each sheep is presented in Table 2.

**Table 1:** Quality of imaging (N = 8 animals) of the relevant anatomical structures during 25 experimental implantations of Impella CP guided by three-dimensional intracardiac echocardiography.

Sheep number	Aortic root	Aortic valve	Left ventricular out flow tract	Left ventricle and papillary muscles	Mitral annulus	Right ventricle
1	10	10	9	8	7	8
2	8	9	8	7	8	10
3	10	8	9	7	9	10
4	8	8	9	6	9	9
5	9	7	9	8	9	9
6	9	9	9	8	8	8
7	10	9	9	9	8	10
8	9	9	8	8	9	10
Value	**9.1 (0.8)**	**8.6 (0.9)**	**8.8 (0.4)**	**7.6 (0.9)**	**8.4 (0.7)**	**9.2 (0.8)**

Values are mean (standard deviation). The quality of imaging was consistent over the three implantations in each sheep (sheep 5 = two implantations, sheep 8 = five implantations).

Significant values are indicated in bold.

**Table 2:** Quality of imaging (N = 8 animals) of the procedural components during 25 experimental implantations of Impella CP guided by three-dimensional intracardiac echocardiography.

Sheep number	Stiff guidewire	Diagnostic catheter	Soft guidewire	Impella catheter	Inflow (Colour Doppler)	Outflow (Colour Doppler)
1	9	9	7	9	7	10
2	7	8	5	8	6	9
3	6	8	4	9	6	10
4	7	8	6	9	8	10
5	8	9	5	8	6	8
6	8	8	7	10	6	9
7	9	9	5	9	7	10
8	8	9	4	10	8	10
Value	**7.8 (1)**	**8.5 (0.5)**	**5.4 (1)**	**9 (0.7)**	**6.8 (0.8)**	**9.5 (0.7)**

Values are mean (standard deviation). The quality of imaging was consistent over the three implantations in each sheep (sheep 5 = two implantations, sheep 8 = five implantations).

Significant values are indicated in bold.

**Figure 1:**
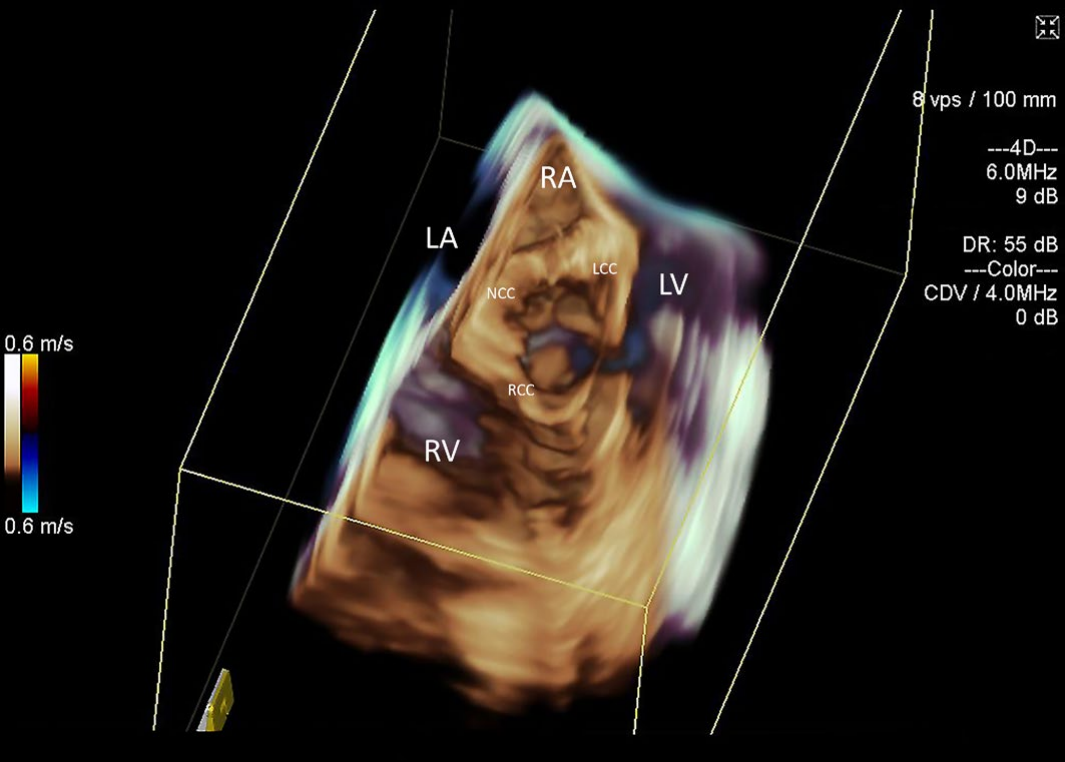
3D ICE *en-face* view of the aortic valve for pre-implantation assessment. *RA* right atrium, *LA* left atrium, *RV* right ventricle, *LCC* left coronary cusp, *RCC* right coronary cusp, *NCC* non-coronary cusp.

**Figure 2:**
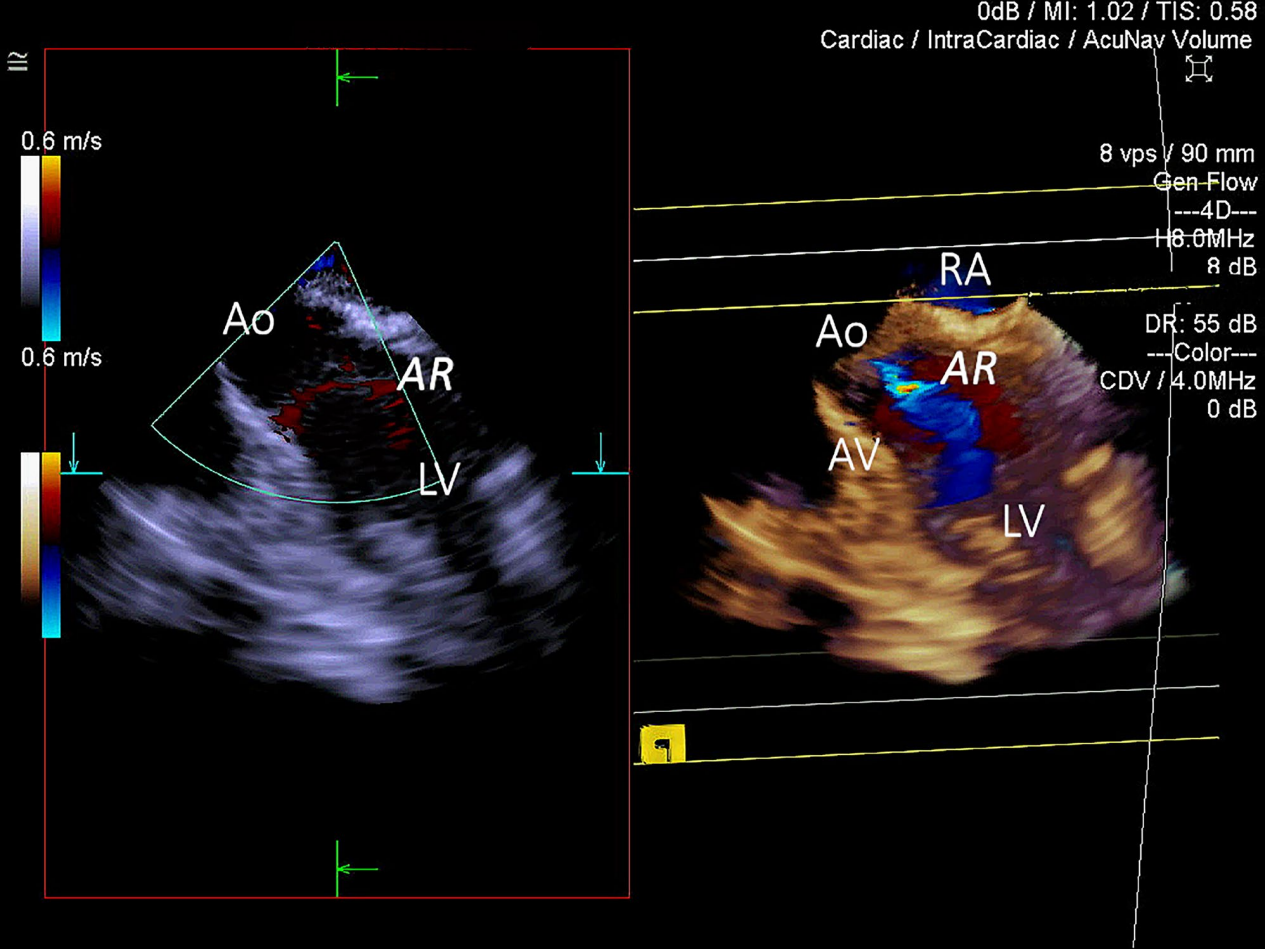
3D ICE Colour Doppler (CD) assessment of the aortic valve for pre-existing pathology. Left side of the image—a cut plane equivalent to the 2D ICE long-axis view of the left ventricle including left ventricular outflow tract, aortic valve and aortic root. Right side of the image—3D CD volume with default cut plane activated. 2D cut plane failed to detect aortic valve insufficiency. *RA* right atrium, *Ao* ascending aorta, *AV* aortic valve, *LV* left ventricle, *AR* trivial aortic regurgitation identified before implantation of the Impella.

**Figure 3:**
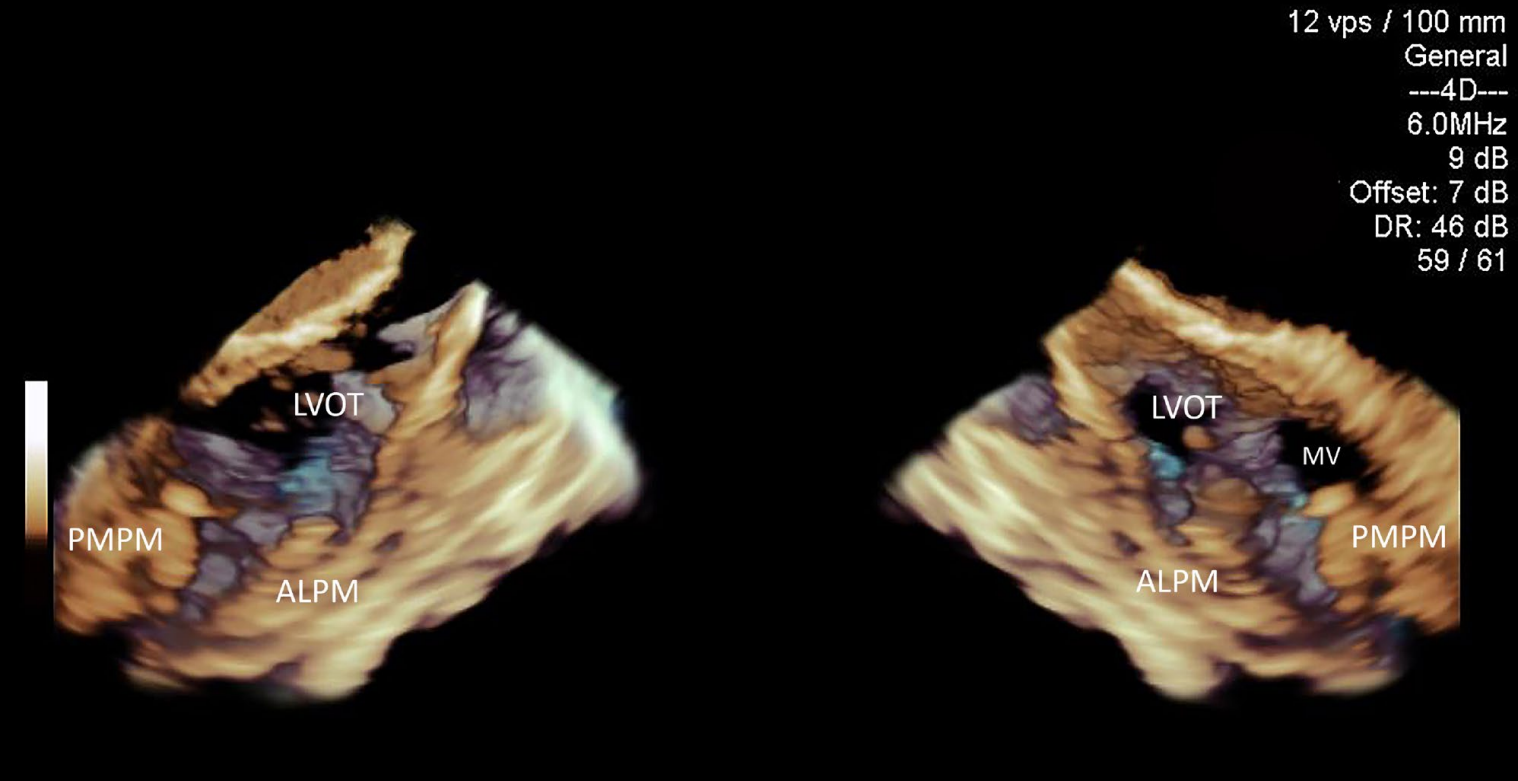
Split open full volume 3D ICE acquisition offers comprehensive long-axis left ventricular image. *LVOT* left ventricular outflow tract, *ALPM* anterolateral papillary muscle, *PMPM* posteromedial papillary muscle, *MV* mitral valve.

**Figure 4:**
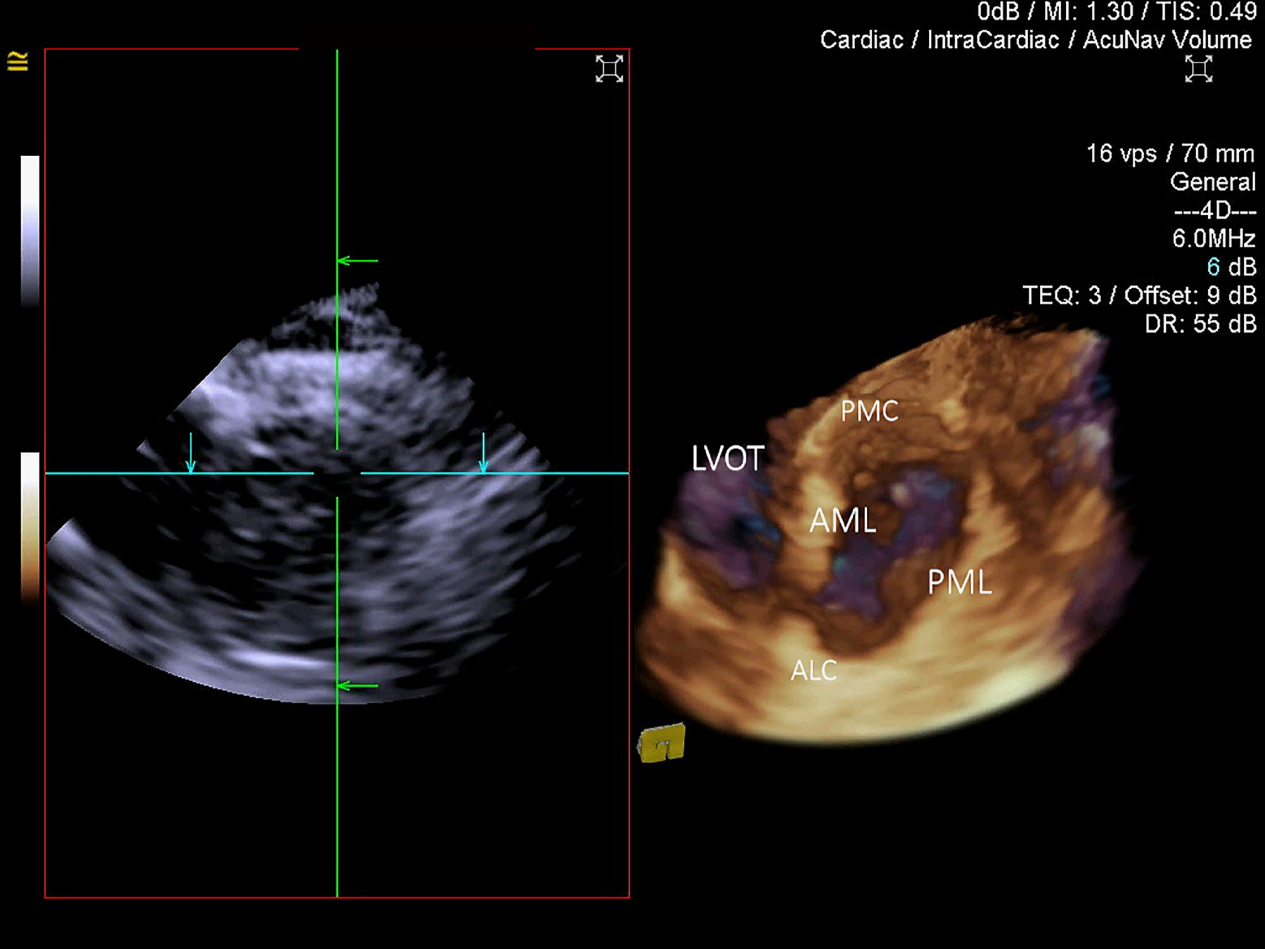
3D ICE en-face ventricular view of the mitral annulus and mitral valve. *LVOT* left ventricular outflow tract, *AML* anterior mitral leaflet, *PML* posterior mitral leaflet, *ALC* anterolateral commissure, *PMC* posteromedial commissure.

Three-dimensional *en-face* images of the aortic valve (Fig. 1, Movie 1) offered delineation of the three leaflets and assessment of their motion to exclude pre-existing aortic valve pathology. The quality of AV assessment was found to be adequate in all cases. The aortic root and proximal ascending aorta were imaged from the right atrial position of the 3D ICE catheter in all cases. Significant intracardiac or aortic anatomic abnormalities were not found in any animals. Activation of the three-dimensional Colour Doppler for assessment of aortic valve incompetence did not reveal underlying pathology beyond trivial aortic regurgitation in two animals (Fig. 2, Movie 2). Advancement of the catheter further into right atrium provided views of the left ventricular outflow tract and left ventricular cavity including both papillary muscles (Fig. 3, Movie 3). The 2D and 3D cut-plane views offered long-axis views of the left ventricle. Minor adjustments in catheter position with retroflexion (posterior steering tilt) were often required to optimize imaging of the mid-and apical portions of the left ventricle. This view offers potential identification of left ventricular thrombus. Anticlockwise rotation opened the ventricular view of the mitral annulus (Fig. 4, Movie 4) and *en-face* view of the mitral valve in all cases.

**Figure 5:**
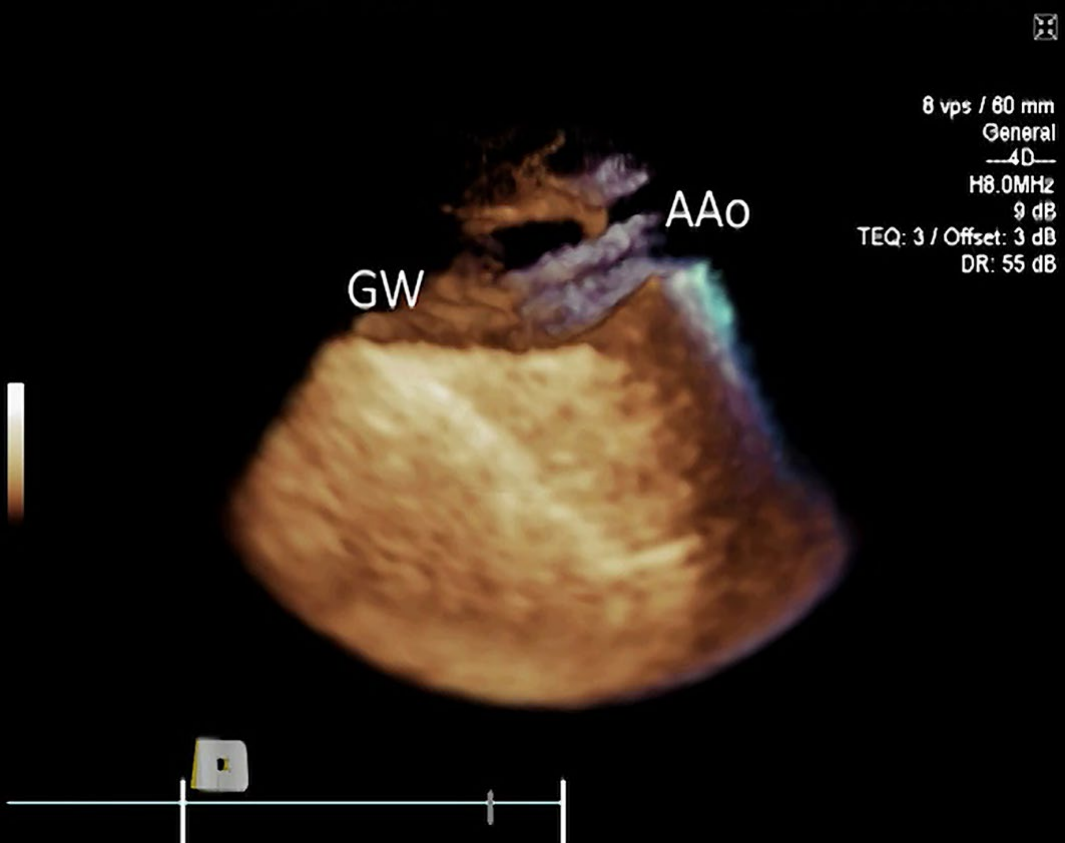
3D ICE image of the ascending aorta (AAo) with Impella CP guidewire (GW).

The J-tip 0.035-inch stiff access guidewire was visible in all cases within the aortic root and ascending aorta (Fig. 5, Movie 5). The guidewire spontaneously entered the ascending aorta in all 8 sheep during all 25 implantations. A J-wire loop forming in the aortic root in one case was immediately identifiable on both 2D and 3D imaging. The position of the cut-plane in 3D ICE volume had to be at times adjusted to include lateral parts of the left ventricular cavity for better visualization of the wire. The wire was clearly identified by 2D ICE, but fan-like rotational manipulations of the ICE catheter were required to place the wire within 2D plane. The spatial relationship between the wire and cardiovascular structures was significantly better appreciated with 3D ICE in all cases. However, some tilting of the 3D volume on the screen was helpful for identification of the wire within left ventricular cavity due to reverberation artifacts. Some reduction in dynamic range settings was helpful for sharper and faster wire visualisation. Inappropriately high gain hindered identification of the wire, especially when it was positioned in a proximity and parallel to the walls of the left ventricle.

**Figure 6:**
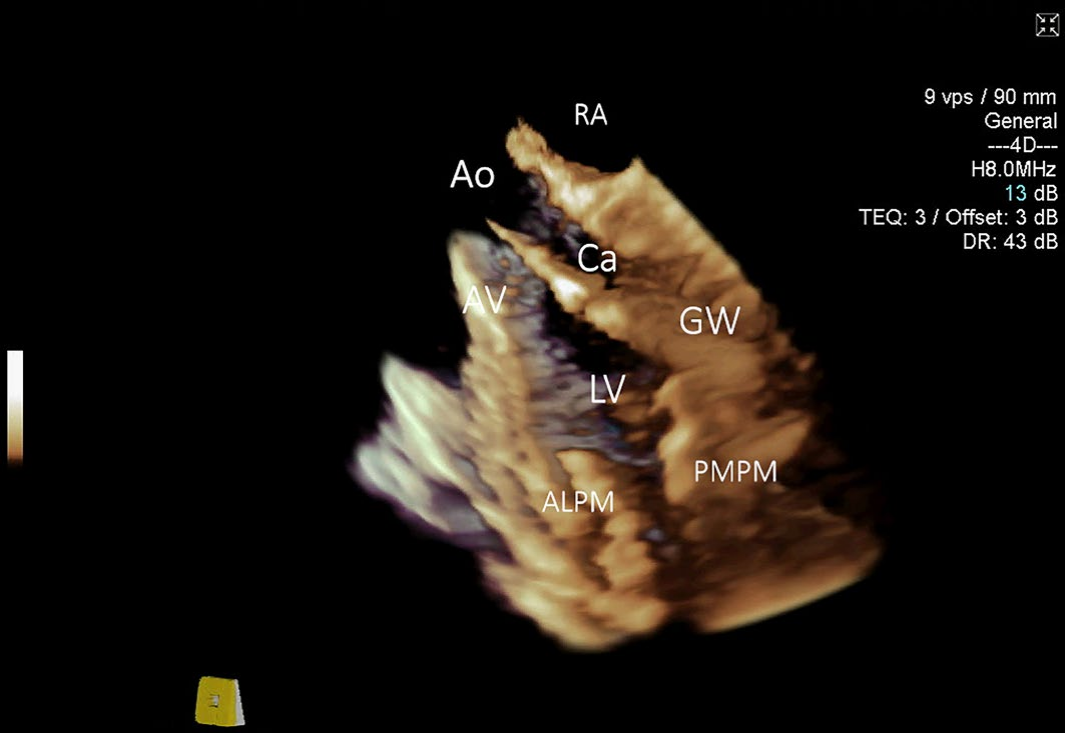
3D ICE full volume imaging with the cut-plane activated along left ventricular (LV) long axis, including aortic valve (AV) and proximal ascending aorta (Ao). *RA* right atrium. Both papillary muscles are clearly delineated. *ALPM* anterolateral papillary muscle, *PMPM* posteromedial papillary muscle. The Impella guidewire (GW) and diagnostic catheter loaded on the guidewire (Ca) is demonstrated traversing the aortic valve, entering LV and terminating near the PMPM. Significant side-lobe artifact makes guidewire appearance unnaturally thick.

**Figure 7:**
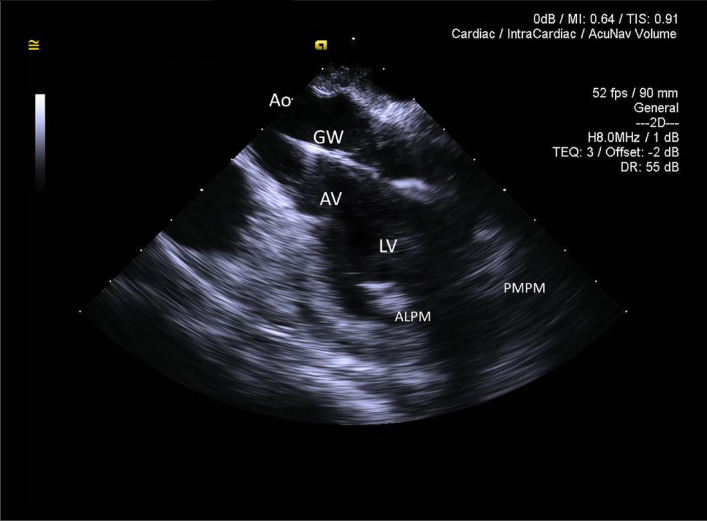
2D ICE long-axis image of the ascending aorta (Ao), aortic valve (AV) and left ventricle (LV). Both papillary muscles are imaged (*ALPM* anterolateral papillary muscle, *PMPM* posteromedial papillary muscle). The Impella guidewire (GW) is seen traversing aortic valve and entering left ventricular cavity. The tip of the guidewire is off-plane, creating false impression that the guidewire is terminating in mid-LV cavity.

**Figure 8:**
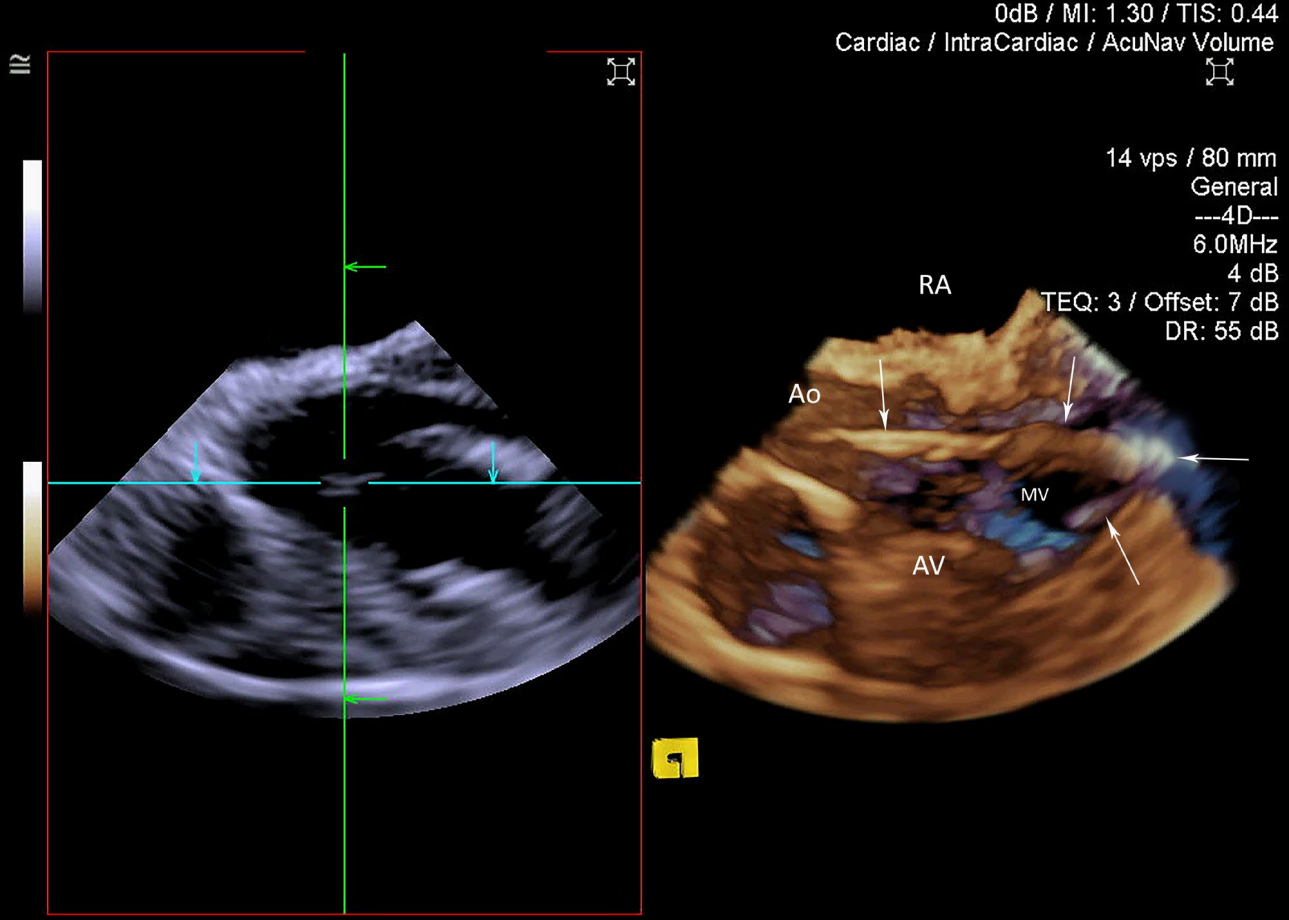
3D ICE full volume imaging with the standard cut-plane activated (left part of the image). The cut-plane is positioned in a middle of the Cartesian space allowing for only partial visualisation of the diagnostic Impella catheter. Free-plane cropping on the right side of the image is simultaneously positioned to allow good assessment of the aortic root, aortic valve (AV), left ventricular outflow tract, basal and mid-segments of the left ventricle. Part of the mitral valve (MV) is seen on the background. The catheter is entering left ventricle via aortic valve, bends and retrograde enters the mitral valve (white arrows) propagating into left atrium. Repositioning was undertaken based on these findings.

A diagnostic catheter was inserted over the stiff guidewire and was also clearly identifiable on 3D ICE in all cases and did not require 2D ICE imaging for clarification. It appeared slightly more echogenic than the wire when compared side by side, but not significantly different when imaged by itself. A side-lobe artifact arising from the guidewire and to the lesser degree from the catheter was noted on several instances (Fig. 6, Movie 6). 2D ICE was sufficient to demonstrate the diagnostic catheter traversing the aortic valve and entering the LV cavity. However, the position of the catheter tip was not always clearly visible (Fig. 7) with 3D ICE providing significantly better positional visualisation (Movie 7). The use of 3D ICE identified catheter malposition retrogradely entering the left atrium via the mitral valve prompting repositioning of the catheter in two sheep (Fig. 8).

Thin soft 0.018-inch guidewire was more difficult to visualise in most cases with inconsistent identification of the forming loops, especially with 3D ICE.

**Figure 9:**
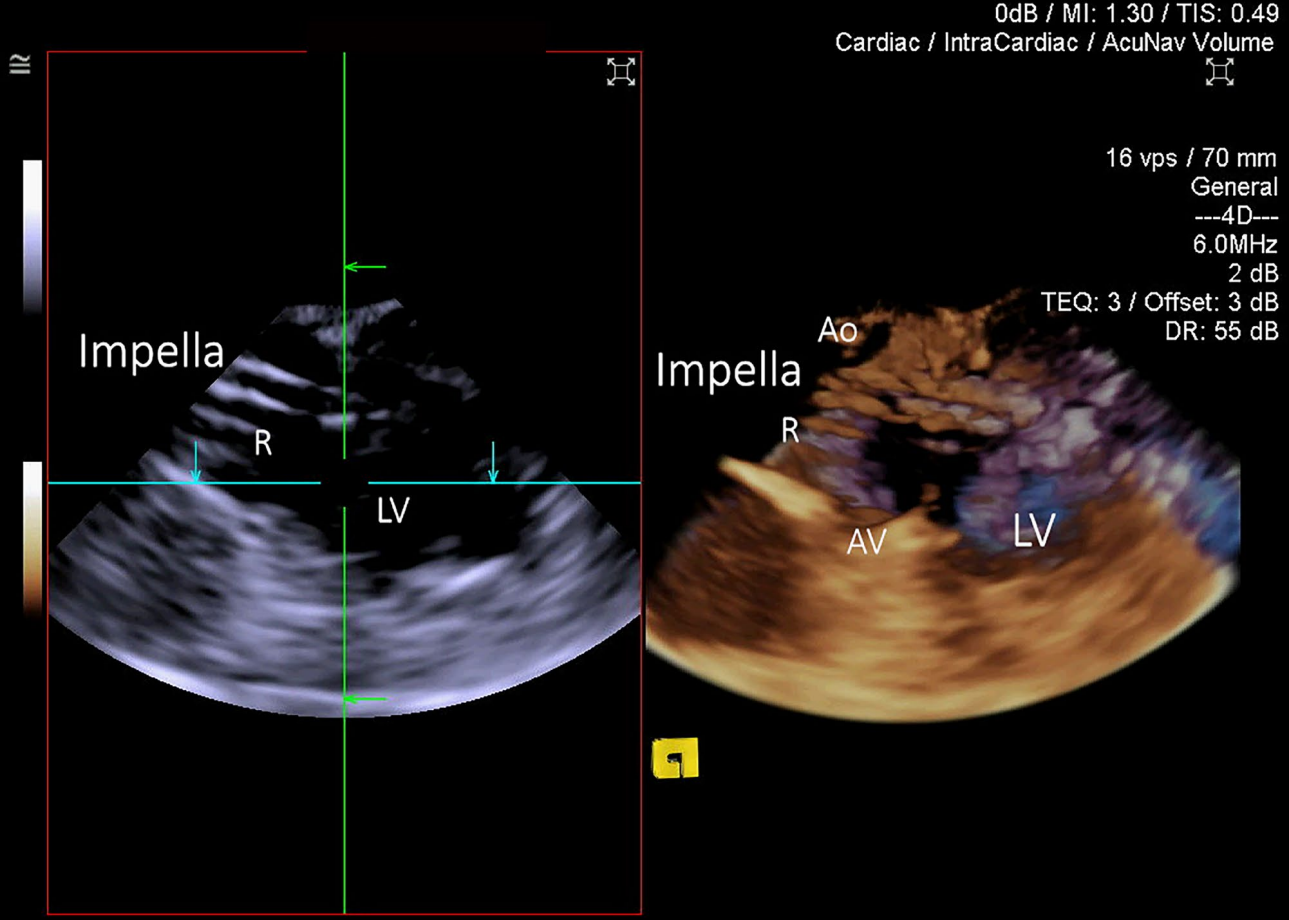
3D ICE image of the Impella catheter entering left ventricle (LV) via aortic valve (AV) from the ascending aorta (Ao). Standard 2D cut-plane image on the left and 3D full volume on the right. Longitudinal cut-plane at the level of the AV presents the Impella CP catheter as a double-walled structure. It is wire-reinforced catheter, which produced significant reverberation artifacts (R).

**Figure 10:**
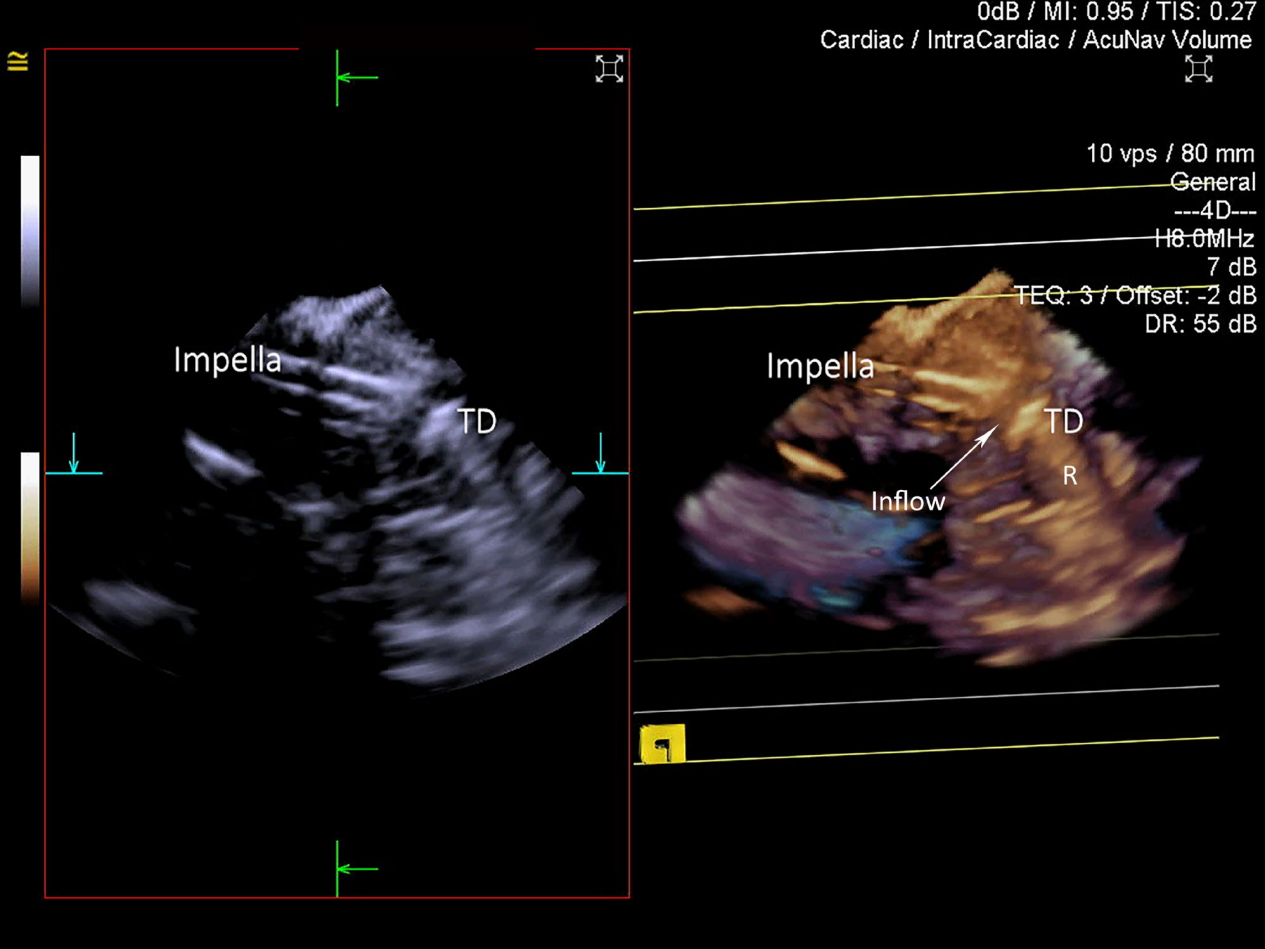
3D ICE image of the Impella CP within LV cavity. The catheter appears as a double-walled structure, while the metal teardrop (TD) cap has highly echogenic structure, causing severe reverberation artifact (R), making visualisation of the plastic J-tip nearly impossible. The teardrop was used to identify the inflow portion of the catheter (arrow) on grey-scale 3D imaging.

**Figure 11:**
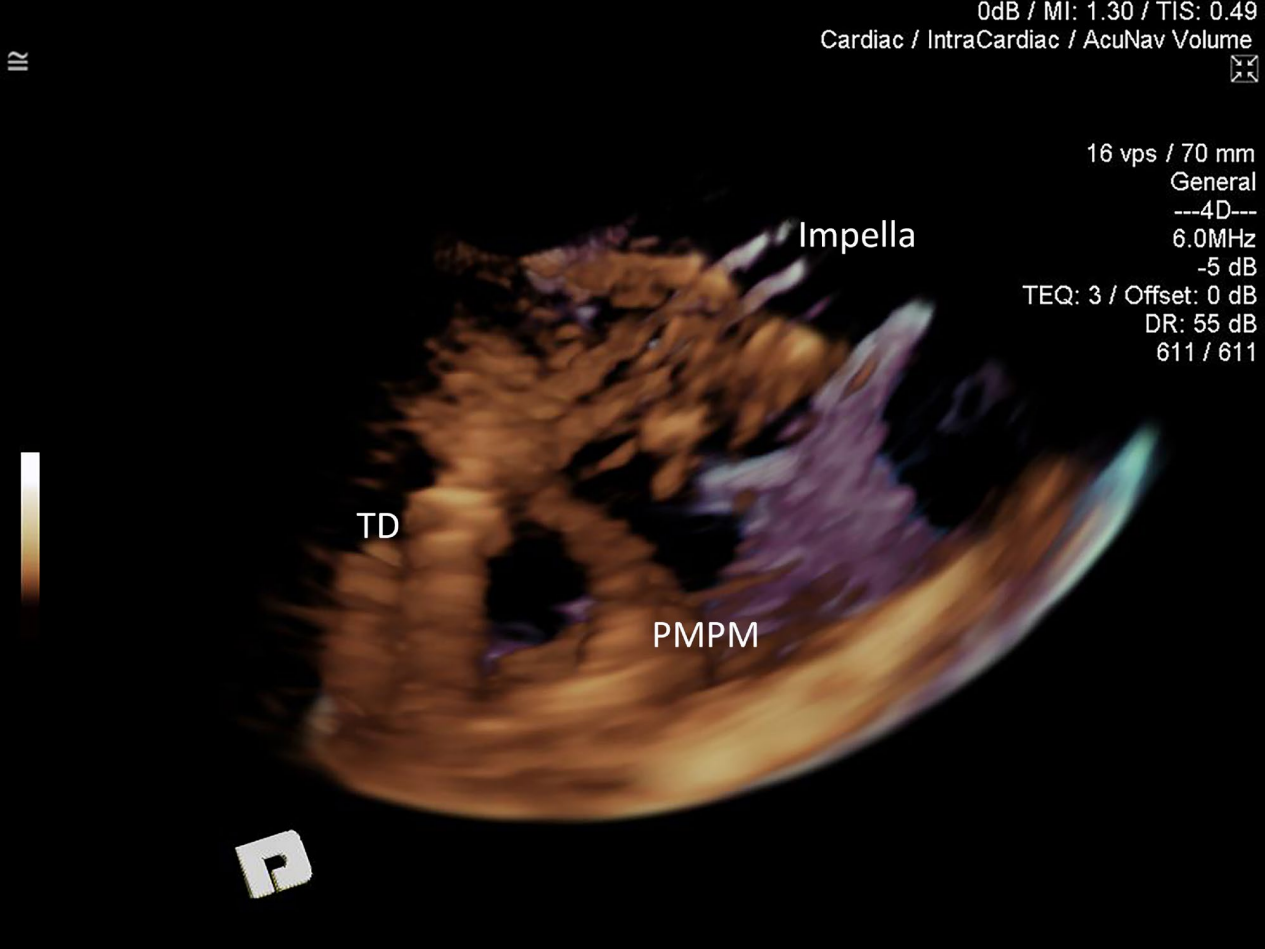
3D ICE. The volume has been rotated to expose the posterior portion of the left ventricle. The Impella CP catheter is well visualised within the left ventricle with the teardrop (TD) found between the posteromedial papillary muscle (PMPM) and left ventricular wall. The inflow portion of the catheter is therefore resting on the tip of the PMPM, amongst the chordal apparatus of the mitral valve. Due to the inadequate flows and high risk of complications, the Impella was repositioned.

The advancement of the Impella pump over the guidewire into the ventricle could be visualized during all Implantations with minor requirement for adjusting the cut-plane position, and occasional rotational adjustment of the ultrasound probe. The reinforced catheter of the Impella correctly appeared in the image as a double-walled structure with cut-plane positioned along the catheter. It created prominent reverberation artifact (Fig. 9, Movie 8). The tear-drop appearance of the metal cap between blood inlet area and plastic pigtail was highly echogenic, making it an ideal 3D marker for positioning (Fig. 10, Movie 9). The Impella plastic pigtail was difficult if not impossible to visualise in all cases. Strong reverberation artifact arising from the tear-drop metal cap at the inflow further complicated visualisation of the plastic pigtail. The relationship between the Impella CP catheter inflow portion of the system and surrounding cardiac structure was superior with 3D ICE when compared to 2D ICE in all cases. However, superior spatial and temporal resolution of 2D imaging offered better appreciation of the aortic valve leaflets and mitral subvalvular apparatus in relation to the Impella. One of the insertions identified the tip of the catheter being impacted under the posteromedial papillary muscle prompting repositioning with slight withdrawal of the catheter (Fig. 11). In 8 implantations the tear drop initially could not be clearly identified due to the impaction into the apex. Slow gradual withdrawal of the Impella under 3D ICE guidance was undertaken until tea-drop became obvious in the mid-LV-cavity.

Overall, the image quality of highly relevant for Impella implantation cardiac structures with 3D ICE was good to excellent (scored 8.6 on a scale 0–10). The quality of imaging components of the procedural equipment with 3D ICE was good (scored 7.8 on a scale 0–10) with exception of thin soft guidewire, which allowed for barely adequate quality.

**Figure 12:**
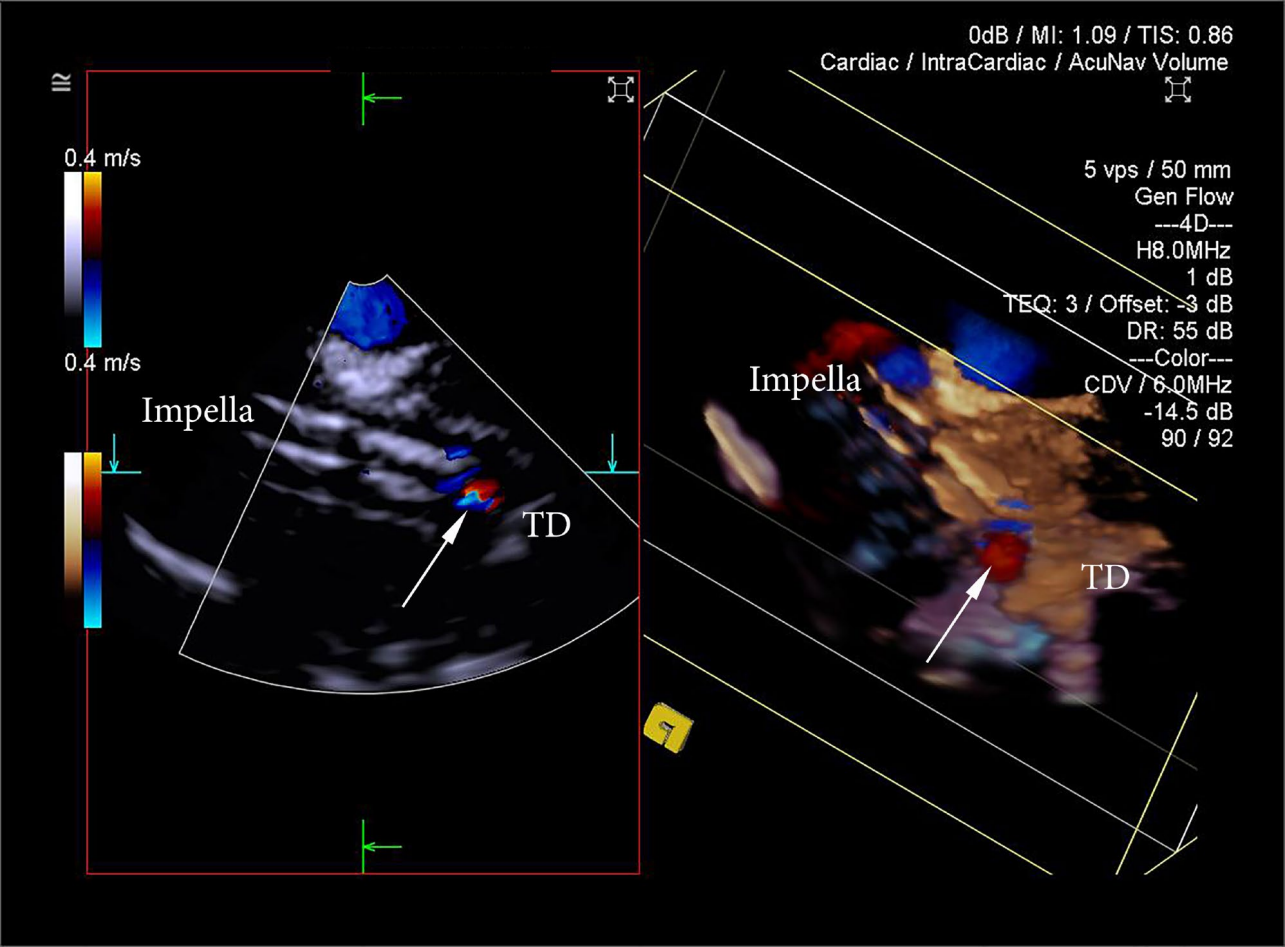
3D ICE Colour Doppler. The colour gain was significantly reduced to minimise interference from intraventricular flows and turbulence associated with Impella. The inflow was identified as an area near tear-drop (TD) on grey-scale imaging and confirmed with present inflow by Colour Doppler (white arrow).

3D Colour Doppler produced extensive “colour bleeding” artifact at the standard settings and required significant reduction in colour gain to identify the blood inlet in the catheter (Fig. 12). Identification of the inlet on 3D Colour Doppler was achieved in 18 implantations and served as an additional confirmation of correct tear-drop identification and inflow site relative position to the cardiac structures. The “3.5 cm” rule applicable for human adults for the position of inflow below aortic valve was not used due to the different anatomical characteristics of the ovine left ventricle. ICE was sufficient in all cases to ensure adequate flows and absence of inflow obstruction as detected by Impella Controller.

The outflow could be visualized in all cases as an extensive turbulence within the proximal ascending aorta above the aortic root. Reduction in colour gain diminished colour “bleeding” over the myocardial tissue in all cases and offered good confirmation of the pump outflow position within the aorta.

**Figure 13:**
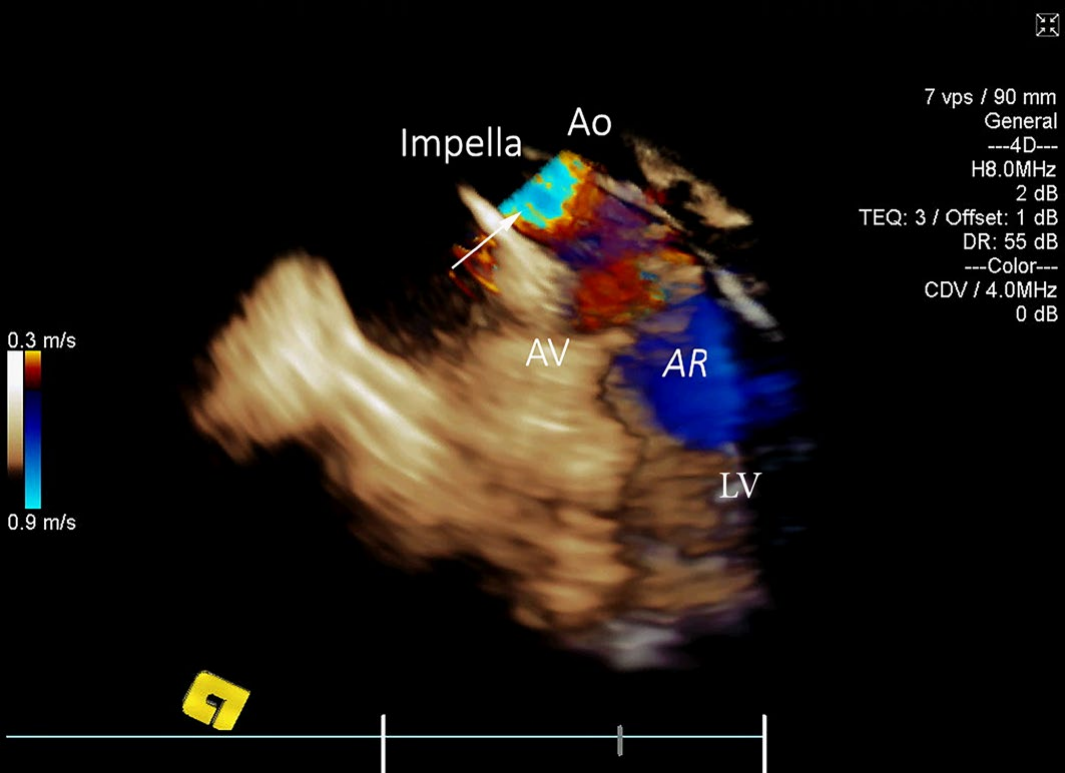
3D ICE Colour Doppler applied over the proximal ascending aorta (Ao), aortic valve (AV) and left ventricular outflow tract. Highly turbulent flow (white arrow) confirms the position of Impella CP outflow. Mild peri-catheter aortic incompetence (AR) was noted. L*V *left ventricle.

3D Colour Doppler was used to reassess the aortic valve for potential incompetence following implantation of the Impella CP. Mild and moderate peri-catheter aortic regurgitation was easily identified on five occasions (Fig. 13, Movie 10).

Postprocessing with adjustments in dynamic range, gain, and colour priority and transparency for each individual image presents better visualization and appreciation of spatial relationships between the catheter and cardiovascular structures.

### Complications

Insertion of the guidewire and the diagnostic catheter inside of the left ventricle frequently caused ectopic cardiac beats due to the direct myocardial irritation by the devices.

There were two failures of the Impella implantation to achieve pump flow:

*Case 1* The guidewire became tangled around intra-aortic pressure catheter and could not be removed after insertion of the pump across the aortic valve. It occurred in sheep 2 during the third implantation. Both 2D ICE and 3D ICE failed to identify the cause of the problem. Removal of the Impella demonstrated severely kinked soft guidewire.

*Case 2* The excessive portion of the guidewire within left ventricular cavity became kinked and tangled around the Impella catheter and could not be removed after insertion of the pump across the aortic valve. An attempt by the operator to apply extra pulling pressure on the guidewire resulted in kinking of the Impella catheter approximately 10 cm above the pump motor housing causing perforation of the carotid artery and resulting in catastrophic bleeding. It occurred in sheep 5 during the second implantation. Both 2D ICE and 3D ICE failed to identify the cause of the problem.

In one sheep the ICE probe entered the right internal thoracic vein and was identified by unusual imaging with the view resembling right ventricular-centric apical transthoracic echocardiographic view. Multiple attempts at repositioning of the probe were required before it was successfully negotiated into the right atrium. This complication occurred in sheep 3 during the first implantation and is likely to be idiosyncratic to sheep.

## Discussion

This translational interventional study demonstrated feasibility of three-dimensional intracardiac echocardiography with a wide azimuthal and elevation of 50° to provide adequate guidance for intracardiac implantation of the Impella CP percutaneous temporary left ventricular assist device in an ovine model. The combination with two-dimensional intracardiac echocardiography modality further improved the quality of imaging and the precision of guidance for optimal Impella CP positioning.

The Impella CP provides a minimally invasive option for temporary left ventricular support. This potentially includes sufferers from COVID-19 cardiac injury with severe left ventricular failure in the absence of respiratory failure, unresponsive to pharmacological therapy^5^. Early mechanical cardiac support should be considered in these patients who have a reasonable probability of recovery, to avoid multiple organ dysfunction resulting from a low cardiac output state.

The optimal choice of the extracorporeal circulatory assist device remains controversial in wide clinical practice and is not yet clear for COVID-19 sufferers. Physiological considerations suggest that in some patients with isolated cardiac complications, left ventricular assist device-based approaches may be superior to VA ECMO in unloading left ventricle while in others a modular approach by combination of both techniques (ECPELLA)^6^ or by combination of VV ECMO and Impella^7^ may offer the best results.

A significant subgroup of COVID 19 patients reportedly developed rapidly progressive cardiac failure during the recovery phase from acute respiratory failure^10^. These patients remain highly contagious, while being unstable with regard to both respiratory and hemodynamic parameters. Transfer of these patients to the catheterisation laboratory for fluoroscopic-guided implantation of the Impella would represent a high risk for these patients, hospital staff and other patients. High oxygen requirements, tachypnea and non-invasive ventilatory support can often be prohibitive for adequate TTE image acquisition^11^. TEE in these patients would require general anaesthesia, reintubation and return to invasive ventilation. This also presents a risk of disease transmission to attending physicians. All the above could be prohibitive for the provision of mechanical cardiac support to otherwise suitable patients. Point of care ICE is the most invasive echocardiographic modality but it could provide an alternative approach to guide awake implantation of the Impella CP under local anaesthesia in this subgroup of patients and avoid undesirable intrahospital transfers and the need for invasive ventilation. The risks associated with ICE must be carefully weighed against the risks of other available imaging modalities within specifics of each individual patient and treating institution.

Although it would be of interest to compare ICE to TTE and TEE, such a comparison was beyond the scope of this study for the following reasons. Our ovine model was thoracotomized for a parallel study making TTE impossible at the time of Impella implantation. The proximity of the right atrium to the aortic root is similar in sheep and humans but the sheep left ventricular apex is midline and the main chamber of the left atrium is lateral to the midline. Previous investigations suggested limited anatomical feasibility of TEE in ovine models^12^. We therefore do not believe that a comparison between intracardiac echo and either TEE or TTE in sheep would be directly applicable to humans. Thus, the study was not designed to compare different echocardiographic techniques but aimed to explore the hypothesis that ICE can be used as a feasible alternative to TTE and TEE when those techniques are clinically unsuitable. Considering translational nature of this study, further human investigation is warranted.

Two-dimensional ICE has been reported in six patients during percutaneous coronary interventions supported by the Impella Recover LP 2.5^13^. ICE was used to assess aortic root and aortic valve, left ventricular outflow tract, left ventricle and mitral apparatus prior to the implantation of the Impella. The ICE probe was inserted via the femoral vein to obtain views from the right atrium. As the ICE was performed in the catheterisation laboratory, standard fluoroscopic guidance may have been used to assist in implantation of the pumps in these patients. 2D ICE was further reported to be used in one patient during percutaneous coronary intervention supported by the Impella Recover LP 2.5. 2D ICE was used in that case for dynamic assessment of short axis left ventricular area from the right ventricular window^14^.

Femoral venous access is normally used for intracardiac echocardiography. Jugular two-dimensional ICE catheter access for guiding intracardiac device implantation has been described^15^ in a patient undergoing transcatheter aortic valve replacement. The authors sited two major benefits—good imaging planes for the anatomical structures of interest (ascending aorta, long-axis aortic valve and long-axis left ventricle) and an absence of interference with the operator inserting the prosthetic valve. Our experimental findings support this report. Percutaneous implantation of the Impella CP is routinely performed via femoral arterial approach. The use of jugular access for the ICE has the benefit of eliminating physical interference with the operators inserting the Impella but might not be preferred in awake COVID patients who have their heads enclosed in a protective barrier.

Transthoracic, transesophageal^3^ and epicardial^16^ echocardiography has been used to navigate implantation of various Impella devices. We did not find any animal or human reports that have described implantation of the Impella with guidance purely by ICE, nor any reports of wide-angle three-dimensional echocardiography used to guide and to optimally position the Impella.

**Figure 14:**
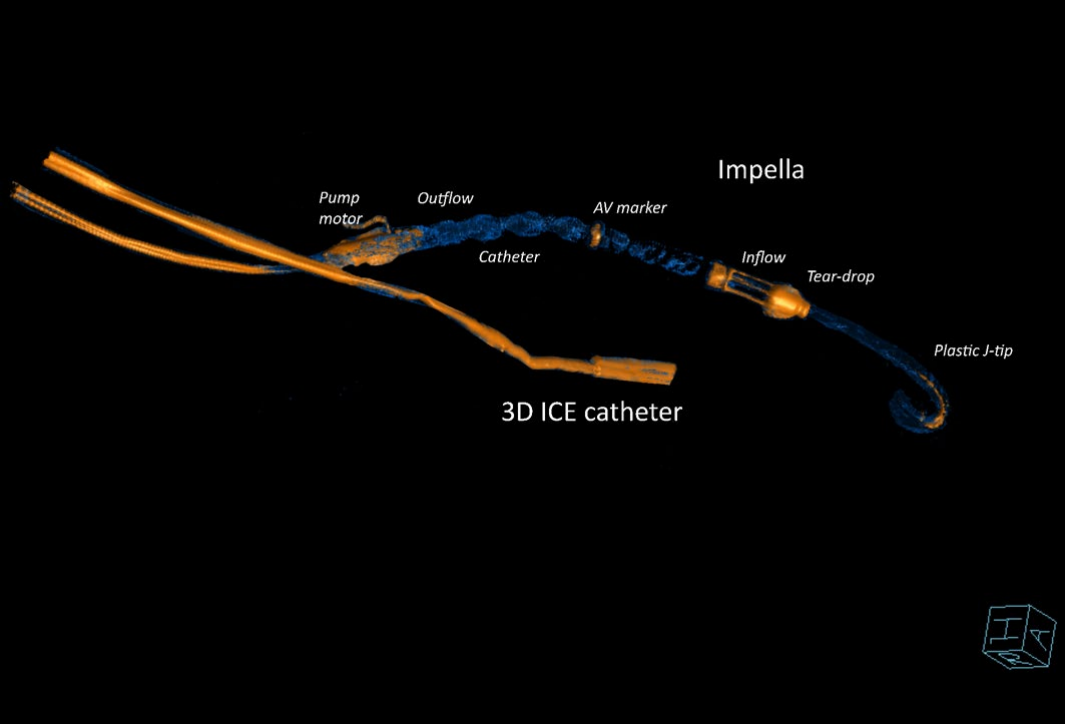
Fluoroscopy with 3D reconstruction has been performed to demonstrate favourable for imaging relationship between the Impella CP implanted into left ventricle and correctly positioned and the 3D ICE catheter with the ultrasound tip located within the right atrium. The proximity to the implanted device allows the use of high ultrasound scanning frequencies, contributing to improved axial spatial resolution. The 2D scanning plane is at a significant angle to the implanted Impella plane, thus requiring significant physical probe manipulations. 3D wide-angle acquisition rectifies this problem by acquiring full volume pyramid of data with elevation of 50° which was sufficient for adequate imaging of the Impella with minimal physical manipulations of the probe when using jugular venous access.

**Figure 15:**
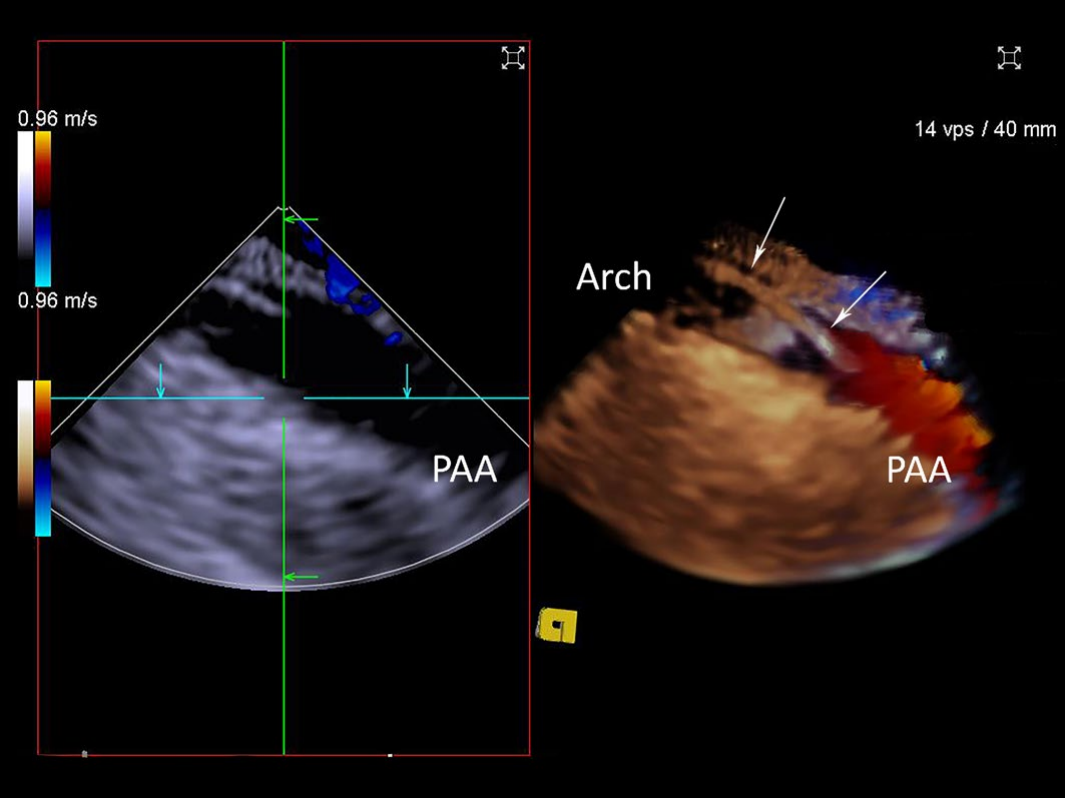
2D ICE (left panel) and 3D ICE Colour Doppler (right panel). Systolic aortic flow is demonstrated in the proximal aortic arch (PAA). The guidewire (white arrows) is clearly visualised within the aortic arch with 3D ICE imaging.

Our intracardiac imaging was adequate in all cases. It required a short learning curve for the sonographer with expertise in 2D and 3D TTE and TEE. Jugular access presented unfamiliar views, but cardiovascular structures relevant to the implantation of the Impella were clearly identifiable. Standard factory scanning settings had to be adjusted for grey scale and Colour Doppler modalities to optimise image quality. We did not use fluoroscopy in any of the cases except to demonstrate the favourable three-dimensional spatial relationship between the implanted pump and ICE probe tip on completion of the insertion and running of the Impella (Fig. 14). Adequate ICE imaging of the ovine aortic arch can be obtained from the superior vena cava to identify a guide wire in the arch (Fig. 15, Movie 11) but such views have not been reported in humans.

Human clinical implantation of Impella CP usually utilize percutaneous femoral or axillary arterial access^13^, with fluoroscopically guided guidewire advancement to the left ventricle. We used a cut-down approach for the left common carotid artery. This approach was selected based on the anatomical specifics of the ovine model and intraoperative convenience and did not imply any recommendations to change traditional human vascular access. The inability of ICE to image the descending aorta has been accepted from the outset as an inherent limitation of this imaging technique where blind guidewire advancement would be required through that portion of the aorta. A similar limitation would apply for TTE, while most of the abdominal aorta would not be imaged with TEE. Guide wires are often inserted into the descending aorta from the femoral artery without any form of imaging as is the case of intra-aortic balloon counter-pulsation. The use of a curved guide wire or sheath dedicated to successful transit of the aortic arch into the ascending aorta will allow blind insertion of the guide wire into the ascending aorta in the majority of patients and can likely be assisted by ICE imaging of the arch. COVID-19 patients potentially selected for the mechanical cardiac support are likely to be younger and without underlying serious vascular pathology, which would minimize the risk of aortic complications during blind introduction of the guidewire through parts of the aorta which are not suitable for imaging with ICE. Careful ultrasound-guided placement of the femoral arterial introducer sheath and high level of postprocedural vigilance in monitoring for potential aortic complications would be justified in cases of “blind” aortic instrumentation. This limitation of ICE constitutes additional risk for the patient and needs to be considered in making clinical decision to select ICE among other imaging techniques.

Two complications with tangled soft guidewire after implantation of the Impella CP across the aortic valve demonstrated important shortcoming of ICE related to the limitations in spatial resolution. In contrast with fluoroscopy, ICE did not clearly identify excessive length of the thin soft guidewire within left ventricle and intravascular trajectory of this guidewire outside Impella between the catheter outflow port and vascular introducer. This led to the unrecognized by echocardiography entanglements and life-threatening complications. Because the guide wire exits the Impella through the pump outflow port, excessive tension on the guide wire is likely to cause bowstringing of the drive sheath resulting in vascular perforation at the point of maximum kinking of the sheath. Accordingly, excessive traction should not be applied to the guidewire. In cases of difficulty during guidewire withdrawal, the whole implanted device together with the Impella CP should be removed as a block.

Limited visualization of the plastic pigtail tip due to the strong reverberation artifact arising from the apex tear-drop part of the Impella CP made it difficult to use this portion of the pump as a guide in one third of experiments. Detection of the tear-drop of the Impella CP impacted into the left ventricular apex suggests a potential danger for left ventricular perforation by the rigid portion of the catheter. Slowing insertion of the device after transiting the aortic valve with ongoing careful dynamic imaging of the tear-drop position instead of the plastic pigtail within left ventricular cavity may help to avoid excessive propagation into the apex and minimize the risk. Vascular perforation occurred in two of the 25 implantations and both were associated with quite forceful traction on the guidewire. The guidewires used were generic rather than those supplied by Abiomed.

The strengths of the study include a pre-specified protocol, high data integrity and simple explorative analysis plan. The study was conducted over a very short inception period of one week considering utmost emergency to develop alternative approaches for COVID 19 patients suffering from acute severe heart failure and the world-wide pandemic closure of most research facilities, including our laboratory.

The limitations of the study include a relatively small sample size. Confounding bias was mitigated by standardising investigative techniques and operator-dependent errors using echocardiography experts for image acquisition and analysis. The translational nature of the study involves utilization of the ovine model thus human application remain speculative.

There have been no previous studies investigating implantation of the Impella CP left ventricular assist device with both two and three-dimensional intracardiac echocardiography.

Our study demonstrates potential to expand the use of the Impella CP for subgroups of patients where other types of imaging to guide implantation are clinically problematic. However, this study acknowledges the invasive nature of ICE with associated potential complications, thus placing the approach into non-standard, reserve category.

## Conclusions

Three-dimensional intracardiac echocardiography is a feasible option to guide implantation of the Impella CP for left ventricular mechanical support and should be carefully considered in cases where other imaging modalities are unsuitable. Clinicians must be aware of the limited human experience, the imaging limitations and the risks associated with this technique in order to avoid serious iatrogenic complications.

## Methods

We conducted a prospective, single-centre translational study of intracardiac echocardiography to guide implantation of the left ventricular assist device (Impella CP) in adult sheep. The study was approved by the University of Sydney (Australia) Animal Research Ethics Committee (2019/1650 amendment) and conducted at the Charles Perkins Centre for Research, The University of Sydney (Sydney, Australia). The study protocol and statistical analysis plan were finalised before data collection was initiated. All methods were performed in accordance with the relevant guidelines and regulations.

### Animals

Animals were acclimatised for at least two weeks prior to the procedure and received routine preventative treatments prior to arrival. Mechanically ventilated, anaesthetised, adult female merino sheep with invasive monitoring of arterial pressure, central venous pressure and cardiac output. Animals received fluid resuscitation and vasopressor support to maintain adequate perfusion pressure. As a part of the parallel study, six animals underwent a left thoracotomy and exposure of the heart prior to the Impella implantations.

### Measurements

All sheep had arterial pressure and central venous pressure monitoring via fluid filled catheters attached to the carotid artery sheath and right internal jugular central venous catheter. A transit time flow probe (Transonic) was placed around the main pulmonary artery via the left thoracotomy. Animals were in the right lateral position with pressure transducers zeroed immediately before the procedure at the level of the right atrium. Mechanical ventilation was conducted in synchronized IPPV mode with PEEP of 5 cm H_2_O. Haemodynamic parameters were continuously recorded.

### Echocardiography

A physician qualified in transthoracic echocardiography (Advanced Transthoracic Echocardiography training, Level 3)^17^ performed all echocardiographic examinations (SC2000, Siemens Healthcare GmbH, Erlangen, Germany). Left internal jugular 14 FG valved cannula was used for the intracardiac probe access. Images were acquired using AcuNav Volume 12.5 F, 90 cm four-ways steerable intracardiac echocardiography catheter (Siemens Medical Solutions, USA Inc, Mountain View, CA). Live three-dimensional volumes were acquired at 6 MHz fundamental and 8 MHz harmonic frequencies with the sector set at 90 × 50° as standard and adjusted together with the depth as required. Maximum achievable frame rate was 60 frames per second for 2D and 14–20 frames per second for 3D grey scale imaging. Temporary resolution was lower for the 3D Colour Doppler, averaging 7–9 frames per second. Right atrial views were obtained with minimal steering for probe positional manipulations, mostly using advance and withdrawal and rotational repositioning. Right ventricular views were not required for the purpose of the study. Echocardiographic quantification was not required. Images underwent cropping and postprocessing using on-cart software. The quality of imaging with three-dimensional intracardiac echocardiography was rated by the expert echocardiographer on an arbitrary scale of 0–10 with the score values applied as following:

0–1—inadequate image quality.

2–3—very poor image quality.

4–5—poor image quality.

5–6—just adequate image quality.

7–8—good image quality.

9–10—excellent image quality.

### Impella CP

A specialist veterinarian surgeon performed implantation of the Impella CP as per manufacturer recommendations, using cut-down approach for the left common carotid artery to provide vascular access. Briefly, the 14Fr 13 cm peel-away introducer was inserted into the left common carotid artery. A stiff 0.035-inch access guidewire was introduced into ascending aorta and then into the left ventricle via aortic valve under ICE guidance. A 6Fr diagnostic catheter was introduced over the wire and advanced into left ventricle under ICE guidance, with repositioning when malposition was identified by echocardiography. The 0.035-inch guidewire was removed and replaced with softer 0.018-inch placement guidewire. The diagnostic catheter was removed and the position of the guidewire within left ventricle confirmed with ICE. The guidewire was loaded in the Impella CP which was then advanced via the carotid sheath into the aorta. The Impella was negotiated over the guide wire through the aortic valve into left ventricular cavity under ICE imaging. The guidewire was removed and the Impella position adjusted by visualizing the tear-drop portion of the catheter in the mid-cavity. An Automated Impella Controller was used to confirm the aortic pressure waveform and the flow started at 1.5 L/min. Colour Doppler confirmation of the inflow and outflow areas was used to readjust position if required. Once the adequate position was confirmed, the flow was increased to 3 L/min to ensure achievement of unimpeded flows.

Following confirmation of normal function, the Impella pump was stopped at the console and then removed completely from the sheep. The process was repeated twice more to account for three implantations in each sheep (the sheep number eight underwent five implantations).

### Statistical analyses

Descriptive statistics were used. Values were expressed as mean (standard deviation) or median [interquartile range].

## Supplementary information

Supplementary Tables.

Supplementary Movie 1.

Supplementary Movie 2.

Supplementary Movie 3.

Supplementary Movie 4.

Supplementary Movie 5.

Supplementary Movie 6.

Supplementary Movie 7.

Supplementary Movie 8.

Supplementary Movie 9.

Supplementary Movie 10.

Supplementary Movie 11.

Supplementary Movie Captions.
