# Publisher Correction: Implantation of Impella CP left ventricular assist device under the guidance of three-dimensional intracardiac echocardiography

**DOI:** 10.1038/s41598-021-82890-1

**Published:** 2021-02-25

**Authors:** Konstantin Yastrebov, Laurencie Brunel, Hugh S. Paterson, Zoe A. Williams, Innes K. Wise, Christopher S. Burrows, Paul G. Bannon

**Affiliations:** 1 Sydney Imaging, Core Research Facility, The University of Sydney, Sydney, Australia; 2 The Baird Institute of Applied Heart and Lung Surgical Research, Sydney, Australia; 3 Institute of Academic Surgery, Royal Prince Alfred Hospital and University of Sydney, Sydney, Australia; 4 Prince of Wales Hospital, Sydney, University of New South Wales, Barker St, Randwick, 2031 Australia

Correction to: *Scientific Reports* 10.1038/s41598-020-74220-8, published online 15 October 2020

This Article contained an error in the labelling of Supplementary Movie files.

Supplementary Movies 11, 1, 2, 3, 4, 5, 6, 7, 8, 9 and 10 were incorrectly published as Supplementary Videos 1, 2, 3, 4, 5, 6, 7, 8, 9, 10 and 11, respectively.

This has now been corrected in the HTML; the PDF version of the paper was correct from the time of publication.

