# Supplementary material for: Implantation of Impella CP left ventricular assist device under the guidance of three-dimensional intracardiac echocardiography
Source: Sci Rep. 2020 Oct 15;10:17485. doi: 10.1038/s41598-020-74220-8 (PMC7562902; doi:10.1038/s41598-020-74220-8)
Supplement: Supplementary file 1 — Supplementary Tables. [file 41598_2020_74220_MOESM1_ESM.docx]





**Implantation of Impella CP left ventricular assist device under the guidance of three-dimensional intracardiac echocardiography.**

Konstantin Yastrebov, Laurencie Brunel, Zoe Williams, Innes Wise, Christopher Burrows, Hugh Paterson, Paul Bannon

**Online data supplement**

**Table E1.** Animals characteristics (N=8) used for 25 experimental implantations of Impella CP. Values are mean (standard deviation) or median [interquartile range] unless indicated otherwise.

| **Sheep number** | **Number of experimental**  **implantations of**  **Impella CP®** | **Age (Years)** | **Sex** | **Body weight (kg)** | **Haemoglobin level (g/dl)** |
| --- | --- | --- | --- | --- | --- |
| **1** | 3 | 2 | Female | 45 | 9.9 |
| **2** | 3 | 2 | Female | 45 | 9.8 |
| **3** | 3 | 2 | Female | 45 | 9.6 |
| **4** | 3 | 2 | Female | 50 | 9.0 |
| **5** | 2 | 2 | Female | 50 | 8.6 |
| **6** | 3 | 2 | Female | 55 | 7.6 |
| **7** | 3 | 2 | Female | 42 | 8.5 |
| **8** | 5 | 1 | Female | 45 | 12.1 |
| **Value** | **Total 25** | **1.9 (0.3)** | **Female (100%)** | **47.1 (3.9)** | **9.4 (1.3)** |

**Table E2.** Haemodynamic and respiratory characteristics (N=8 animals) prior to the implantations of the Impella CP. Values are mean (standard deviation) or median [interquartile range].

| **Sheep number** | **Heart rate (bpm)** | **Systolic blood pressure (mm Hg)** | **Diastolic blood pressure (mm Hg)** | **Mean arterial pressure (mm Hg)** | **Central venous pressure (mm Hg)** | **Hemoglobin oxygen saturation (%)** |
| --- | --- | --- | --- | --- | --- | --- |
| **1** | 92 | 72 | 65 | 68 | No record | 95 |
| **2** | 115 | 79 | 49 | 60 | 12 | 87 |
| **3** | 82 | 81 | 60 | 69 | No record | 94 |
| **4** | 73 | 97 | 78 | 85 | 12 | 94 |
| **5** | 86 | 95 | 78 | 85 | 7 | 97 |
| **6** | 77 | 93 | 70 | 81 | 4 | 98 |
| **7** | 84 | 89 | 67 | 75 | 12 | 95 |
| **8** | 118 | 104 | 79 | 89 | 14 | 70 |
| **Value** | **91 (16)** | **89 [80-97]** | **69 [61-78]** | **78 [68-85]** | **10 (3.5)** | **91 (8.6)** |
