# Supplementary material for: Implantation of Impella CP left ventricular assist device under the guidance of three-dimensional intracardiac echocardiography
Source: Sci Rep. 2020 Oct 15;10:17485. doi: 10.1038/s41598-020-74220-8 (PMC7562902; doi:10.1038/s41598-020-74220-8)
Supplement: Supplementary file 13 — Supplementary Movie Captions. [file 41598_2020_74220_MOESM13_ESM.docx]

**Movie 1.**

3D ICE *en-face* view of the aortic valve for pre-implantation assessment. RA – right atrium, LA – left atrium, RV – right ventricle, LCC – left coronary cusp, RCC – right coronary cusp, NCC – non-coronary cusp.

**Movie 2.**

3D ICE Colour Doppler (CD) assessment of the aortic valve for pre-existing pathology. Left side of the image – a cut plane equivalent to the 2D ICE long-axis view of the left ventricle including left ventricular outflow tract, aortic valve and aortic root. Right side of the image – 3D CD volume with default cut plane activated. 2D cut plane failed to detect aortic valve insufficiency. RA – right atrium, Ao – ascending aorta, AV - aortic valve, LV – left ventricle, AR – trivial aortic regurgitation identified before implantation of the Impella.

**Movie 3.**

Split open full volume 3D ICE acquisition offers comprehensive long-axis left ventricular image. LVOT – left ventricular outflow tract, ALPM – anterolateral papillary muscle, PMPM – posteromedial papillary muscle, MV – mitral valve.

**Movie 4.**

3D ICE en-face ventricular view of the mitral annulus and mitral valve. LVOT – left ventricular outflow tract, AML – anterior mitral leaflet, PML – posterior mitral leaflet, ALC – anterolateral commissure, PMC – posteromedial commissure.

**Movie 5.**

3D ICE image of the ascending aorta (AAo) with mobile Impella CP guidewire (GW).

**Movie 6.**

3D ICE full volume imaging with the cut-plane activated along left ventricular (LV) long axis, including aortic valve (AV) and proximal ascending aorta (Ao). RA – right atrium. Both papillary muscles are clearly delineated. ALPM – anterolateral papillary muscle, PMPM – posteromedial papillary muscle. The Impella guidewire (GW) and diagnostic catheter loaded on the guidewire (Ca) is demonstrated traversing the aortic valve, entering LV and terminating near the PMPM. Significant side-lobe artifact makes guidewire appearance unnaturally thick.

**Movie 7.**

3D ICE full volume imaging demonstrated excellent view of the Impella CP diagnostic catheter traversing the aortic valve (AV) from the aorta (Ao), entering left ventricle (LV) and impacting into the left ventricular apex next to the base of the anterolateral papillary muscle (ALPM).

**Movie 8.**

3D ICE image of the Impella catheter entering left ventricle (LV) via aortic valve (AV) from the ascending aorta (Ao). Standard 2D cut-plane image on the left and 3D full volume on the right. Longitudinal cut-plane at the level of the AV presents the Impella CP catheter as a double-walled structure. It is wire-reinforced catheter, which produced significant reverberation artifacts (R).

**Movie 9.**

3D ICE image of the Impella CP within LV cavity. The catheter appears as a double-walled structure, while the metal teardrop (TD) cap has highly echogenic structure, causing severe reverberation artifact R), making visualisation of the plastic J-tip nearly impossible. The teardrop was used to identify the inflow portion of the catheter (arrow) on grey-scale 3D imaging.

**Movie 10.**

3D ICE Colour Doppler applied over the proximal ascending aorta (Ao), aortic valve (AV) and left ventricular outflow tract. Highly turbulent flow (white arrow) confirms the position of Impella CP outflow. Mild peri-catheter aortic incompetence (AR) was noted.

**Movie 11.**

3D ICE imaging obtained from the superior vena cava, demonstrating the guide wire (GW) traversing mid-aortic Arch and descending to the proximal aortic arch (PAA).
